# 2020 Update to Spinal Muscular Atrophy Management in Saudi Arabia

**DOI:** 10.3389/fped.2021.684134

**Published:** 2021-05-31

**Authors:** Fahad A. Bashiri, Mohamad-Hani Temsah, Khalid Hundallah, Fahad Alsohime, Yazed AlRuthia

**Affiliations:** ^1^College of Medicine, King Saud University, Riyadh, Saudi Arabia; ^2^Division of Pediatric Neurology, Department of Pediatrics, King Saud University Medical City, Riyadh, Saudi Arabia; ^3^Pediatric Intensive Care Unit, Pediatric Department, King Saud University Medical City, Riyadh, Saudi Arabia; ^4^Division of Neurology, Department of Pediatrics, Prince Sultan Military Medical City, Riyadh, Saudi Arabia; ^5^Department of Clinical Pharmacy, College of Pharmacy, King Saud University, Riyadh, Saudi Arabia; ^6^Pharmacoeconomics Research Unit, College of Pharmacy, King Saud University, Riyadh, Saudi Arabia

**Keywords:** spinal muscular atrophy, gene therapy, nusinersen, onasemnogene abeparvovec, risdiplam, Saudi Arabia

## Abstract

Novel therapeutic strategies have shown some promise in treating spinal muscular atrophy (SMA). However, the outcomes and acceptance of these new strategies are yet to be explored. We aimed to investigate physicians' opinions and perceptions toward management strategies of SMA across Saudi Arabia. This is a cross-sectional survey using a self-administered, structured questionnaire sent to physicians who care for SMA patients during the Saudi Pediatric Neurology Society annual conference. A total of 72 clinicians of different neurological subspecialties were included. 48.6% prescribed nusinersen to their patients, with 39% of them having patients started on nusinersen. Though, 8.3% prescribed onasemnogene abeparvovec for 1–3 patients, while none of their patients started on the treatment. 64.3% stated that the only treatment available for SMA in their settings is supportive care. Around 69.4% described having a moderate to high knowledge on SMA gene therapy, and 79.2% would recommend it. 48.6% confirmed they would prescribe gene therapy at the age of 6 months, and 78.3% would prescribe it for type-I SMA. Pediatric neurologists are receptive to novel and innovative therapies for SMA in Saudi Arabia. However, the high treatment acquisition cost, strict regulations, logistical issues, and budget constraints delay their adoption and implementation.

## Introduction

Spinal muscular atrophy (SMA) is a major autosomal recessive neuromuscular disorder (1). SMA causes motor neuron degeneration in the brain stem and spinal cord, leading to progressive muscle weakness and atrophy (2). Although it is rare, with an estimated incidence rate of 4–10 per 100,000 newborns (3–7), it has the highest mortality rate among affected children compared to other genetic diseases (8).

The SMA pathophysiology is believed to be due to mutations in the survival motor neuron 1 (SMN1) gene (9), which most commonly lead to deletions, in turn causing a deficiency of SMN (10). Low levels of full-length functioning SMN (~10%) can partly compensate for SMN1 deficiency (11). All SMA patients have at least one copy of SMN2 (12). They are categorized into SMA type 1–4 (SMA1–SMA4), depending on the age at which the disease started and their motor activity (13). In addition, the number of SMN2 copies is inversely related to the severity of the disease phenotype (14). The frequency of the carrier that causes SMN1 mutations ranges from 1 in 90 to 1 in 47 (5–7).

The standard of care in SMA is mainly supportive and consists of nutritional and respiratory support, as needed, in addition to management of muscle weakness complications (13, 15–17). There have been many recent developments in the field of SMA treatment (18). Nusinersen (Spinraza®), onasemnogene abeparvovec (Zolgensma®), and risdiplam (Evrysdi®) are three innovative disease-modifying drugs approved by multiple regulatory bodies, such as the US Food and Drug Administration (USFDA) and the European Medicines Agency (EMA).

SMA patients are deficient in SMN. Nusinersen is a synthetic antisense oligonucleotide that promotes exon 7 inclusion to promote increased SMN production. Nusinersen was approved by the USFDA in December 2016 to manage both pediatric and adult SMA patients (19). It also received marketing authorization approvals in Europe, Japan, Brazil, Canada, and Australia (20).

Another approach to treating SMA is gene replacement of the mutated SMN1 with normal SMN1. Onasemnogene abeparvovec is an adeno-associated viral vector-based gene therapy drug that replaces the function of the missing or non-functioning SMN1 gene into the patient's cells. It is a once-only treatment given by an intravenous infusion over 60 min. In May 2019, it became the first gene therapy drug for SMA to be approved by the USFDA. Onasemnogene abeparvovec is indicated for the treatment of children <2 years old who have bi-allelic SMN1 mutations (21). In May 2020, the European Commission (EC) granted a conditional drug approval (22).

Risdiplam was recently approved by the USFDA and is indicated for the treatment of children aged 2 months or more (23). It is a small-molecule SMN2-splicing modifier. It is orally administered, and it reaches the central nervous system as well as peripheral organs, increasing the production of full-length SMN mRNA (24, 25).

One of the “problems of medical care for SMA patients” is that the decision-making regarding SMA management in pediatric patients largely depends on the treating neurologists' and institutions' experiences or small-scale experimental and/or observational studies (15). Despite emerging evidence of the safety and efficacy of the newly approved SMA therapies (26), their acceptance among neurologists in Saudi Arabia is still unclear. This study explored the perceptions and attitudes of SMA-treating neurologists and other specialties toward the use of novel and innovative treatment options for SMA management in Saudi Arabia.

## Subjects and Methods

### Study Design

This online, questionnaire-based, cross-sectional study was conducted between February 27 and 29, 2020, among licensed neurologists who attended the annual national Saudi Pediatric Neurology Society Conference. The inclusion criterion was neurologists who provided care for SMA patients, who consented to participate in the survey, and who filled out the questionnaire. A panel of experts in the field of neurology analyzed the questionnaire for its content, and adjustments were made based on feedback from five academic experts. A total of 72 neurologists filled out the questionnaire, and their responses were considered for analysis.

### Data Collection

The questionnaire comprised 25 items including questions on the respondents' sociodemographic and professional characteristics, patient demographic data, current SMA management plans, and perceived obstacles to the adoption and use of new treatment options for SMA management in Saudi Arabia. In addition, the respondents were asked about their awareness and attitude toward gene therapy for SMA management.

### Ethical Considerations

The study was approved by the institutional review board of the College of Medicine, King Saud University (E18-3539). All information about the participating neurologists was kept confidential.

### Statistical Analysis

We used means and standard deviations to describe continuous variables and frequencies and percentages to describe categorical variables. We performed the Kolmogorov–Smirnov and Levene's tests to assess normality and homogeneity assumptions, respectively, of continuous variables. All statistical analyses were conducted using SPSS® Statistics (IBM Corporation, Armonk, NY, USA).

## Results

### General Characteristics of Respondents

A total of 72 neurologists filled out the questionnaire. Of the 72 respondents, 43 (59.7%) were males, 63.9% were consultants, and 63.9% specialized in pediatric neurology. The years of experience varied, with 45.8% having 1–5 years of experience, with the mean being 8.4 (6.9) years. In addition, 65.3% of the respondents worked in tertiary care hospitals. Table 1 lists Respondents' sociodemographic and professional characteristics.

**Table 1:** Responders' sociodemographic and professional characteristics.

**Characteristics**	**Frequency (*N*)**	**Percentage (%)**
**Sex**		
Male	43	59.7
Female	29	40.3
**Clinical role**		
Consultant	46	63.9
Specialist	10	13.9
Resident	16	22.2
**Main field of practice**		
Pediatric neurology	46	63.9
Adult neurology	5	6.9
Pediatric intensive care	14	19.4
Pediatrics	4	5.6
Genetics	3	4.2
**Experience years categorized**		
1–5 years	33	45.8
6–10 years	19	26.4
≥11 years	20	27.8
**Working Healthcare facility type**		
Tertiary hospital	47	65.3
Secondary hospital	23	31.9
Primary health care	2	2.8

The respondents were asked a set of questions to describe their experience with SMA patients. More than half of the respondents (54.2%) saw <5 SMA patients per year.

The respondents were also asked about three SMA treatments: nusinersen (Spinraza®), onasemnogene abeparvovec (Zolgensma®), and risdiplam (Evrysdi®). Thirty five (48.6%) of the respondents prescribed nusinersen, with 39% having their patients started on nusinersen, while ~8.3% prescribed onasemnogene abeparvovec to 1–3 patients, none of whom were taking.

In addition, the respondents were asked about the main obstacles to obtaining these three drugs. The most common reason reported was internal hospital logistics (47.1%), followed by logistical issues outside the hospital (44.3%). In addition, 40.3% of the respondents said that the new medications changed the SMA patients' hospital code. With regard to current treatment options, ~64.3% of the respondents said that full supportive care is the only treatment option in their hospitals, while 31.4% reported that palliative care, do-not-resuscitate, and no endotracheal intubation are the current therapies in their hospitals (Table 2).

**Table 2:** Practices and experience of physicians with SMA patients.

**Practices and experience**	**Frequency (*N*)**	**Percentage (%)**
**How many cases of SMA do you treat per year?**		
>5	39	54.2
5–10 cases per year	21	29.2
more than 10 per year	3	4.2
I do not treat any SMA patient currently	9	12.5
**For how many patients did you prescribe nusinersen?**		
None	37	51.4
1–3 patients	13	18.1
4–6 patients	14	19.4
**How many of these patients did they start on nusinersen?**		
None	44	61.1
1–3 patients	18	25
4–6 patients	6	8.3
≥7 patients	4	5.6
**For how many patients did you prescribe onasemnogene abeparvovec?**		
None	66	91.7
1–3 patients	4	5.6
4–6 patients	2	2.8
**How many of these patients did they start on onasemnogene abeparvovec?**		
None	72	100
**What were the main obstacles you had in obtaining these SMA treatments?**		
Hospital internal/Related reasons	33	47.1
Logistics reasons outside my hospital	31	44.3
Saudi FDA approval	8	11.4
Financial/cost	39	55.7
Family-related obstacles	6	8.6
Other reasons	10	7.9
**Did the new treatments (like nusinersen or gene therapy) change your hospital code status for SMA patients?**		
No	43	59.7
Yes	29	40.3
**In your hospital, what are the current treatment options for SMA?**		
Full supportive care	45	64.3
Non-invasive ventilation	21	30
Tracheostomy and home ventilation	20	28.6
Palliative care, do-not-resuscitate, and no endotracheal intubation	22	31.4
Others	5	7.1

*SMA, spinal muscular atrophy*.

### Knowledge and Attitude Toward Gene Therapy for SMA Treatment

The respondents were asked about their knowledge of and attitude toward gene therapy and its use to treat SMA. About 69.4% of the respondents described their knowledge of gene therapy for SMA as moderate to high, and 79.2% said that they would recommend gene therapy for SMA in their area, 48.6% said that they would prescribe gene therapy for SMA patients as young as 6 months, and 78.3% said that they would prescribe it for Type-I SMA.

The respondents were also asked about the main obstacles to not using gene therapy for SMA. About 73.9% of the respondents said that the major obstacle was the high cost of medication, while 55.1% said it was the lack of expertise with novel, innovative therapies.

With regard to the hospitals' preparedness to use gene therapy for SMA, 41.7% of the respondents said that their hospitals were not prepared for this type of treatment, while only 8.3% found their hospitals entirely prepared for initiating gene therapy once it becomes available (Table 3).

**Table 3:** Knowledge and attitude toward gene therapy for SMA.

**Knowledge and attitude toward gene therapy for SMA**	**Frequency (*N*)**	**Percentage (%)**
**Describe your current knowledge of gene therapy and its use in treating neuromuscular diseases, such as SMA?**		
Very low	6	8.3
Low	16	22.2
Moderate	34	47.2
High	14	19.4
Very high	2	2.8
**Would you recommend gene therapy for treating SMA disease in your region?**		
No	15	20.8
Yes	57	79.2
**In your opinion, what would be the main obstacle(s) for not using gene therapy for SMA patients?**		
Lack of expertise in using gene therapy since it is new	38	55.1
Other available treatment	5	7.2
Price/cost of treatment	51	73.9
Low efficacy	10	14.5
Side effects	2	2.9
Other	7	10.1
**If you are likely to prescribe gene therapy for SMA patients, what would be the recommended age for treating the patient?**		
Below 2 years	23	31.9
Below 1.5 years	4	5.6
Below 1 year	10	13.9
Below 6 months	35	48.6
**If you are likely to suggest gene therapy for SMA patients, what would be the recommended type of SMA?**		
SMA Type-I	54	78.3
SMA Type-II	38	55.1
SMA Type-III	25	36.2
**How prepared is your hospital in terms of Multidisciplinary teams to treat patients with gene therapies such as gene therapy for SMA?**		
Not at all prepared	30	41.7
Just starting to prepare	14	19.4
Making good progress with the preparation	14	19.4
Very nearly prepared	8	11.1
Completely prepared	6	8.3

*SMA, spinal muscular atrophy*.

### Clinical Judgment of Respondents on Different SMA Case Scenarios

The respondents were given two different case scenarios of SMA patients and were asked about their clinical decision in each scenario. The first scenario was of an infant who did not require respiratory support, and the second scenario was of an infant who had respiratory failure and required 12 h intubation. Onasemnogene abeparvovec was the selected treatment option in (55.6 and 33.3% for the two scenarios, respectively); ~50% of the respondents said that they would prescribe onasemnogene abeparvovec because of its efficiency.

In addition, more than 50% of the respondents agreed to using endotracheal intubation for acute exacerbation for SMA patients (51.4%) and to chronic home ventilation for SMA patients (58.3%). On the contrary, 50% of the respondents were against chronically ventilating SMA patients in hospitals. Table 4 lists all responses.

**Table 4:** Responses of the respondents toward the two case scenarios.

**Responses toward the two case scenarios**	**Frequency (*N*)**	**Percentage (%)**
**Scenario-1: A hypotonic 3-month-old infant was admitted to your hospital with a respiratory failure diagnosis secondary to hypoventilation. Genetic testing confirmed SMA. Patient is on room air, with no respiratory support. If all the following treatments are available at your hospital: Which type of treatment would you give this infant?**		
Onasemnogene abeparvovec	40	55.6
Nusinersen	18	25
Risdiplam (once available)	6	8.3
Other supportive treatments	4	5.6
Neither (no SMA modifying medication)	4	5.6
**What is the reason(s) for your choice of therapy in the above question?**		
The efficiency of this treatment	33	50
Availability of the treatment	16	24.2
Other reasons	14	21.2
Neurology team expertise	12	18.2
Price/costs of treatment	9	13.6
Family request	2	3
**Scenario-2: A hypotonic 6-month-old infant was admitted to your hospital with a respiratory failure diagnosis secondary to hypoventilation. Genetic testing proved the infant has SMA. The patient has been intubated 12 h ago. If all the following treatments are available in your hospital, would you give this infant this type of treatment?**		
Onasemnogene abeparvovec	24	33.3
Neither (no SMA modifying medication)	21	29.2
Nusinersen	16	22.2
Risdiplam (once available)	9	12.5
Other supportive treatments	2	2.8
**What is the reason for your choice of therapy in the above question?**		
The efficiency of this treatment	30	47.6
Neurology team expertise	16	25.4
Availability of the treatment	12	19
Price/costs of treatment	9	14.3
Other reasons	9	14.3
Family request	2	3.2
**What is your opinion about the following statement: “in acute exacerbation, endotracheal intubation is acceptable for SMA patients”**		
Agree	37	51.4
Neither agree or disagree	17	23.6
Disagree	18	25
**What is your opinion about the following statement: “chronic home ventilation is acceptable for SMA patients”**		
Agree	42	58.3
Neither agree nor disagree	11	15.3
Disagree	19	26.4
**Do you have chronically ventilated SMA patients in your hospital?**		
Yes	31	43.1
No	36	50
I do not know/Not sure	5	6.9

*SMA, spinal muscular atrophy*.

## Discussion

This study evaluated the level of acceptance and knowledge of the novel, innovative therapies used for SMA management among a sample of clinicians involved in the care of pediatric SMA patients in Saudi Arabia. Several studies have explored the best approaches to SMA management to improve the patients' and their families' quality of life (15, 27–29).

After decades of poor prognosis of SMA, the USFDA recently approved gene therapy for SMA treatment (8, 19–21, 23, 30–33). The new SMA modifying treatments have significantly changed the disease outcome, especially when initiated prior to the emergence of symptoms (34, 35). Our findings indicate that the utilization rate of novel and innovative treatments for SMA management is low in Saudi Arabia (more than 50% of the respondents reported never prescribing nusinersen to their patients). However, most SMA patients who were prescribed nusinersen are taking it. In contrast, nearly all of the respondents did not prescribe onasemnogene abeparvovec, and no patient was started on it because this drug is not yet approved by the Saudi FDA. Our findings also highlight the significant transformation in SMA management in Saudi Arabia because a sizable minority of SMA patients have already started taking nusinersen, which was not available just a few years ago (27). Not surprisingly, two-thirds of the respondents said that only supportive care is available for their patients, and one-third used palliative care, do-not-resuscitate, and no endotracheal intubation, which is in accordance with international consensus guidelines (15, 29).

Onasemnogene abeparvovec for SMA treatment is widely accepted. Hoy et al. (36) reported the milestones of onasemnogene abeparvovec development in Europe and the United States. Onasemnogene abeparvovec is the first gene therapy to be approved for SMA management in children <2 years old (36). Therefore, there is a high preference for and acceptance of onasemnogene abeparvovec among neurologists in Europe and the United States (36). However, onasemnogene abeparvovec is the most expensive drug in the world, making it unaffordable for most healthcare institutions (36). This was clearly indicated by the majority of the respondents, who reported that the high acquisition cost of onasemnogene abeparvovec is the most daunting obstacle to using this novel treatment for their SMA patients and requires Ministry of Health approval. However, early economic reports have indicated better incremental cost-effectiveness ratios (ICERs) of onasemnogene abeparvovec compared to nusinersen despite the significantly higher cost of onasemnogene abeparvovec (37), probably because onasemnogene abeparvovec is administered only once in a lifetime compared to nusinersen, which must be administered regularly (37). Another obstacle is related to the logistics: 8.3% of the respondents said that their hospitals are prepared to handle gene therapy, which is consistent with the literature (38).

With regard to obstacles to the use of gene therapy, 73.9% of our respondents said that the major obstacle is the high cost, followed by a lack of expertise in using gene therapy, hospital logistical, and regulatory considerations. However, according to “SMA Therapies: ICER Grounds the Price to Value Conversation in Facts (37), the ICER quality-adjusted life-year (QALY) cost-effective model for treating SMA found higher gains in QALY and life-years gained for onasemnogene abeparvovec compared to nusinersen.

Although 80% of our respondents had low to moderate knowledge of gene therapy for SMA, they said that they would recommend it. More than 50% of our respondents recommended onasemnogene abeparvovec because of its efficacy. In addition, 48.6% of them said that they would prescribe it for children <6 months old, while 78.3% said that they would prescribe it for SMA1. These data are similar to the healthcare providers' acceptance of other gene therapy modalities.

Al-Zaidy et al. (39) reported that onasemnogene abeparvovec is a promising drug and can achieve early treatment success in young infants with SMA.

To aid the rational use of onasemnogene abeparvovec for the treatment of SMA, a group of European neuromuscular experts published a consensus paper covering qualification, patient selection, safety considerations, and long-term monitoring. In addition, it is important that only qualified neurologists use gene therapy in expert centers (40).

This study had a few limitations. First, the number of pediatric neurologists in Saudi Arabia who are specialized in SMA management is limited. In 2017, the estimated total pediatric neurologist in Saudi Arabia was 75, with a ratio of 1.06 neurologists per 100,000 children (41). Therefore, the SMA management scope in Saudi Arabia might be shared with other specialties, which were not included in this study. Second, like all self-reported studies, the validity of outcomes depended on the respondents' honesty and subjective opinions. Third, involving trainees/residents may have affected some of the responses to questions especially when it come to the obstacles and care of patients with SMA. Finally, the study used convenience sampling in one setting, limiting the generalizability of the findings.

## Conclusion

There is broad acceptance for different, novel, and innovative treatment options, including gene therapy, for SMA. Therefore, the entry of such novel, innovative, and high-cost treatments should be through an outcome-based agreement until the long-term follow-up data confirm real-world effectiveness. In parallel, the physicians' knowledge of these medications should be enhanced. Furthermore, decision-makers in the healthcare sector should consider our findings to facilitate SMA management.

## Data Availability Statement

The raw data supporting the conclusions of this article will be made available by the authors, without undue reservation.

## Ethics Statement

The study was approved by the institutional review board of the College of Medicine, King Saud University (E18-3539). All information about the participating neurologists was kept confidential. The patients/participants provided their written informed consent to participate in this study.

## Author Contributions

All authors listed have made a substantial, direct and intellectual contribution to the work, and approved it for publication.

## Conflict of Interest

Medical writing services were hired by Novartis Gene Therapies that took no part in the analysis or interpretation of the data. The authors declare that the research was conducted in the absence of any commercial or financial relationships that could be construed as a potential conflict of interest.
